# The atomistic origin of the extraordinary oxygen reduction activity of Pt_3_Ni_7_ fuel cell catalysts†
†Electronic supplementary information (ESI) available: Computational details and additional structural and ORR catalytic information on the Pt–Ni de-alloyed particles. See DOI: 10.1039/c5sc00840a


**DOI:** 10.1039/c5sc00840a

**Published:** 2015-04-29

**Authors:** Alessandro Fortunelli, William A. Goddard III, Luca Sementa, Giovanni Barcaro, Fabio R. Negreiros, Andrés Jaramillo-Botero

**Affiliations:** a CNR-ICCOM and IPCF , Consiglio Nazionale delle Ricerche , via Giuseppe Moruzzi 1 , 56124 , Pisa , Italy . Email: alessandro.fortunelli@cnr.it ; Email: afloer@caltech.edu; b Materials and Process Simulation Center (MC 139-74) , California Institute of Technology , Pasadena , California 91125 , USA . Email: wag@wag.caltech.edu

## Abstract

Recently Debe *et al.* reported that Pt_3_Ni_7_ leads to extraordinary Oxygen Reduction Reaction (ORR) activity. However, several reports show that hardly any Ni remains in the layers of the catalysts close to the surface (“Pt-skin effect”). This paradox that Ni is essential to the high catalytic activity with the peak ORR activity at Pt_3_Ni_7_ while little or no Ni remains close to the surface is explained here using large-scale first-principles-based simulations. We make the radical assumption that processing Pt–Ni catalysts under ORR conditions would leach out all Ni accessible to the solvent. To simulate this process we use the ReaxFF reactive force field, starting with random alloy particles ranging from 50% Ni to 90% Ni and containing up to ∼300 000 atoms, deleting the Ni atoms, and equilibrating the resulting structures. We find that the Pt_3_Ni_7_ case and a final particle radius around 7.5 nm lead to internal voids in communication with the exterior, doubling the external surface footprint, in fair agreement with experiment. Then we examine the surface character of these nanoporous systems and find that a prominent feature in the surface of the de-alloyed particles is a rhombic structure involving 4 surface atoms which is crystalline-like but under-coordinated. Using density-functional theory, we calculate the energy barriers of ORR steps on Pt nanoporous catalysts, focusing on the O_ad_-hydration reaction (O_ad_ + H_2_O_ad_ → OH_ad_ + OH_ad_) but including the barriers of O_2_ dissociation (O_2ad_ → O_ad_ + O_ad_) and water formation (OH_ad_ + H_ad_ → H_2_O_ad_). We find that the reaction barrier for the O_ad_-hydration rate-determining-step is reduced significantly on the de-alloyed surface sites compared to Pt(111). Moreover we find that these active sites are prevalent on the surface of particles de-alloyed from a Pt–Ni 30 : 70 initial composition. These simulations explain the peak in surface reactivity at Pt_3_Ni_7_, and provide a rational guide to use for further optimization of improved catalytic and nanoporous materials.

## Abstract 2

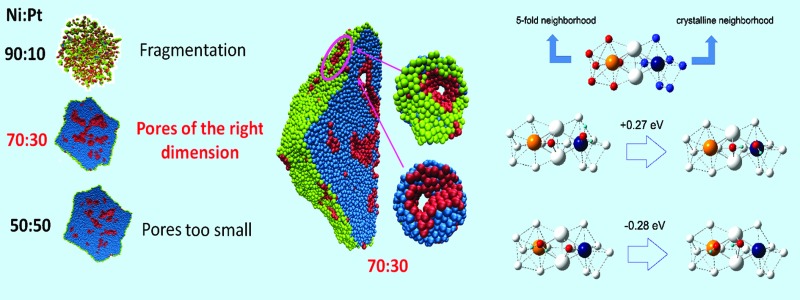
Optimality of Pt : Ni 30 : 70 fully dealloyed nanoporous Pt particles in terms of size and coordination environment.

## 1. Introduction

Currently a major impediment in the utilization of the hydrogen fuel cells essential for sustainable and energy-efficient electrical power generation, is the extremely slow Oxygen Reduction Reaction (ORR),^1^–^3^ which is several orders of magnitude slower than the anode reaction, requiring too much Pt catalyst thereby increasing the cost significantly.^4^,^5^ There has been a great deal of excitement about the use of Pt-based alloys,^1^–^3^ especially the Pt–Ni alloy that is the most promising and active system.^6^–^8^ A particularly interesting observation by Debe *et al.* is that the catalytic activity of Pt–Ni depends strongly on Ni content, exhibiting a maximum at 69 to 73% Ni initial composition.^1^–^3^ This alloy leads to a significant increase in activity with respect to systems of neighboring initial composition under similar conditions. However the origin of the extraordinary activity of the Pt_3_Ni_7_ catalysts is puzzling, since many studies (see *e.g.* Fig. 8(C–E) of ref. 9 for a latest report) find that cycling under fuel cell electrochemical conditions rapidly removes most or all Ni atoms near the surface, thickening then pure Pt surface layers. In the words of ref. 2:

“The sharp dependence of ORR activity on at% Ni at the Pt_3_Ni_7_ composition begs explanation. We believe it is highly unexpected directly from any d-band density or oxygen binding energy calculations, DFT or lattice strain modeling, segregated surface structure or de-alloyed Pt-skeleton, Pt-skin or core–shell structures studied to date.”

In order to elucidate the mechanism by which Pt_3_Ni_7_ could lead to a peak in ORR performance, we report here first-principles-based (ReaxFF) Reactive Molecular Dynamics (RMD) simulations combined with a Local Embedded Cluster Re-optimization (LECR) procedure on de-alloyed Ni–Pt particles with compositions of 50 to 90% Ni. We start from Pt–Ni random alloy nanoparticles with sizes of 3.4 to 10 nm in radius (∼13 000 to ∼330 000 atoms) and simulate the effect of de-alloying by making the extreme assumption of removing all Ni and allowing the Pt structure to readjust and optimize. We demonstrate that alloys near 70% lead to a substantial increase in surface area because of internal voids connected to the surface through large pores and that the surface structure of the de-alloyed Pt nanoporous is atomistically smooth and crystalline-like but under-coordinated compared to normal surfaces, with first-principles calculations showing that this leads to a significant reduction in the energy barrier for the rate-determining step in ORR. These factors thus predict a maximum catalytic activity at around 70% Ni initial composition, in excellent agreement with the experimental evidence. These investigations extend our understanding of ORR catalysts, complementing previous simulations and experiments to explain improved activity.^10^–^12^

## 2. Results

### 2.a The fully de-alloyed Pt–Ni particle hypothesis

One paradox in the field of ORR catalysis by Pt-based alloys consists in the well established fact that alloyed systems can exhibit a catalytic activity significantly enhanced with respect to pure Pt analogues, despite the fact that after a few ORR electrocatalytic cycles the minority element (which is a more electropositive element than Pt such as Ni, Co or Mn) is effectively leached out of the outermost 4–6 surface layers and is transferred into the solution as cationic species. A residual Ni content is left in the core of the particles with an amount varying from few percent to 15–18% depending on the catalyst treatment and the dimensions of the nanosystems.^1^–^9^ Understanding such “Pt-skin” effect and its striking dependence upon Ni initial concentration is extremely challenging. Based on the experiments showing that the specific ORR performance of Pt_3_Ni_7_ is dramatically improved,^1^–^3^ but that post-catalysis characterization finds no Ni close to the Pt_3_Ni_7_ surface,^1^–^9^ we speculated that the Pt_3_Ni_7_ system is fully de-alloyed near the surface. This led us to consider the extreme hypothesis of fully de-alloying the entire particles, which corresponds to *propagating the surface layers devoid of Ni into the bulk*. The surface atomistic configurations thus determined, which are crucial for catalysis, should be close to the experimental ones which are also fully de-alloyed. What is neglected in this hypothesis are: (1) electronic effects due to residual Ni (this seems unlikely, since the residual Ni lie into the bulk), and (2) long-range strain effects due to the lattice mismatch between the Ni–Pt alloyed core and the pure Pt surface,^33^ as discussed in depth in ref. 49. Of course this effect may be damped by the structural freedom especially in the direction perpendicular to the surface. Full de-alloying thus seems an extreme but useful working hypothesis. This approach departs significantly with respect to our previous studies that used QM methods to determine the detailed changes in the compositions of the alloys near the surface and the effects of this on the various steps involved in ORR. Thus we now test a totally different region of the phase diagram than previously investigated (also with respect to other studies, see *e.g.*ref. 50), and determine the consequences on ORR catalytic activity.

To pursue this hypothesis, we considered a series of Pt–Ni nanoparticles for three different particle sizes:

• ∼3.4 nm in radius or 13 000 total atoms,

• ∼8 nm in radius or 180 000 total atoms, and

• ∼10 nm in radius or 330 000 total atoms

each constructed as an fcc truncated-octahedral arrangement with random distribution of Ni and Pt atoms for initial Pt–Ni compositions ranging from 50 : 50 to 10 : 90 in steps of 5%. We then removed all Ni atoms, and relaxed the system using the ReaxFF reactive force field^13^,^14^ to optimize Pt–Pt interactions^15^ (using the LAMMPS^16^ software, see the ESI† for more details).

The nanoparticle geometry after Ni removal was optimized locally and then equilibrated *via* Molecular Dynamics (MD) runs at the typical temperature of fuel cell experiments (70 °C) until no significant structural change was observed (typically, 2 to 7 nanoseconds). To accumulate statistics, we generated 5 initial distributions for each size and composition.

Modeling an experimental corrosion process *via* computational simulations is an extremely difficult task, due to the long time scales and the size of the systems. The protocol here employed, in which Ni removal is followed by local geometry optimization and dynamical relaxation, models in a simplified way the complex de-alloying process in which Ni surface atoms are leached out with the remaining Pt atoms diffusing towards nearby empty sites resulting in new facets and opening physical spaces for pore formation. In our protocol, the Pt atoms that are initially in an fcc-like although randomly sparse framework are able to coalesce into denser structures which keep memory of the original pattern, thus mimicking a kinetics-driven de-alloying process.

In the experiment, the amount of Ni remaining in the catalyst depends on the method of analysis and preparation, ranging from 5 at% to 20 at%,^1^–^8^ with much of this in the interior of the particle, far from the catalytic surface. Indeed in ref. 2 the amount of this residual Ni content element does not depend significantly on the Ni initial composition, and a highly porous Pt-skin type model was proposed to explain the high activity of high Ni-content catalysts, with bulk Ni dissolution produced under ORR conditions creating a high catalytic surface area which undergoes minimal re-arrangement under successive cycling.^17^ This Pt-skin picture has been confirmed in several studies, see *e.g.*ref. 9. Thus to explain the origin of improved performance of NiPt alloys and particularly the performance at peak Pt_3_Ni_7_, we make the extreme assumption of complete Ni removal and use first-principles-based simulations to achieve understanding of the nanoparticle structures and properties as a function of composition and particle size. These calculations allow us to explain all experimental results providing, we believe, considerable insight into the mechanism by which de-alloying enhances ORR catalysis.

### 2.b Structural analysis of de-alloyed particles

**Fig. 1:**
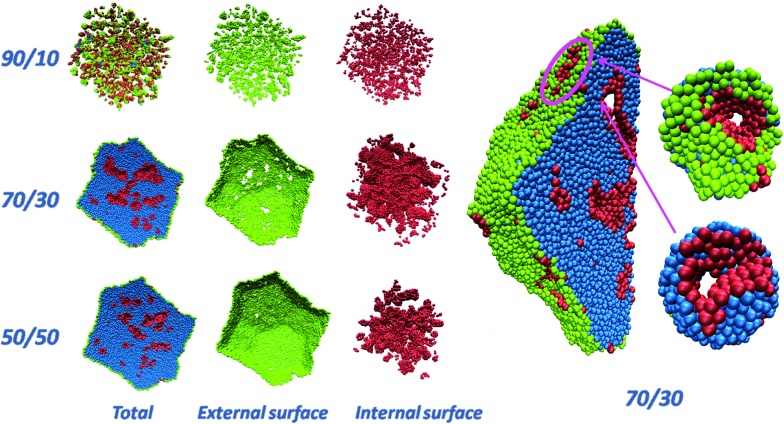
(left, first three columns) Pictorial views of de-alloyed Pt particles obtained starting from truncated-octahedral Pt–Ni nanoparticles about 10 nm in radius with initial compositions Pt–Ni ranging from 50 : 50 to 30 : 70 to 10 : 90) after complete removal of Ni, followed by minimization and molecular dynamics equilibration at 70 °C. For clarity, a cross-section (half) through the Pt particles is shown. “Total” is the entire particle, “External surface” shows the atoms on the external surface of the particle (colored in green). “Internal surface” shows the atoms on the internal nanopores (colored in pink). Inner (core) atoms are colored in cyan (right). Closer views of the initially 10 nm-radius particle with Pt–Ni = 30 : 70 composition after de-alloying, including details of selected nanopores in the insets.

Fig. 1 shows a pictorial example of the atomistic configurations obtained from fully de-alloying a 10 nm-radius particle (330 000 atoms) with three selected compositions. For illustrative purposes, a cross-section (half) through the Pt particles is shown. “Total” is the whole particle, with atoms on the external surface of the particle in green, atoms on the surfaces of the internal nanopores of the particle in pink, and inner (core) atoms in cyan. The “External surface” shows a view of the Pt atoms on the external surface of the particle, while the “Internal surface” shows atoms on the surfaces of the internal nanopores.

Here we distinguish surface and bulk atoms according to the following procedure: for each Pt, its first-neighbors are defined as those atoms lying within a distance of 3 Å (the Pt–Pt bond of bulk Pt is 2.775 Å), then a coordination vector is evaluated as the sum of all the vectors pointing from first-neighbors to the given atom. The given Pt atom is defined as a bulk atom if the norm of the coordination vector is smaller than a given threshold (0.6 Å), and as a surface atom otherwise. A surface atom is further identified as external if – by taking a step of 20 Å along the coordination vector – no other Pt atom is found in a sphere of 10 Å around the end point, otherwise it is identified as an internal atom. From an inspection of Fig. 1 it is immediately apparent that when the initial composition is too rich in Ni (*e.g.*, Pt–Ni = 10 : 90), the final particle comes out fragmented into tiny clusters, whereas if the initial composition is too low in Ni (*e.g.*, Pt–Ni 50 : 50) nanocavities exist within the particle but they tend to “close up” without reaching the surface (hence, they are inaccessible to reactants). Instead, for an initial composition around Pt–Ni = 30 : 70, percolating nanopores^18^ that reach up to the surface are found throughout the particle. For example, at Pt–Ni = 30 : 70 initial composition (or Pt_3_Ni_7_) the diameter of the nanopores is ≈9 Å, which is large enough to contain ≈3H_2_O layers to allow for O_2_ diffusion. The Cartesian coordinates of the particles shown in Fig. 1 are provided as ESI.†

**Fig. 2:**
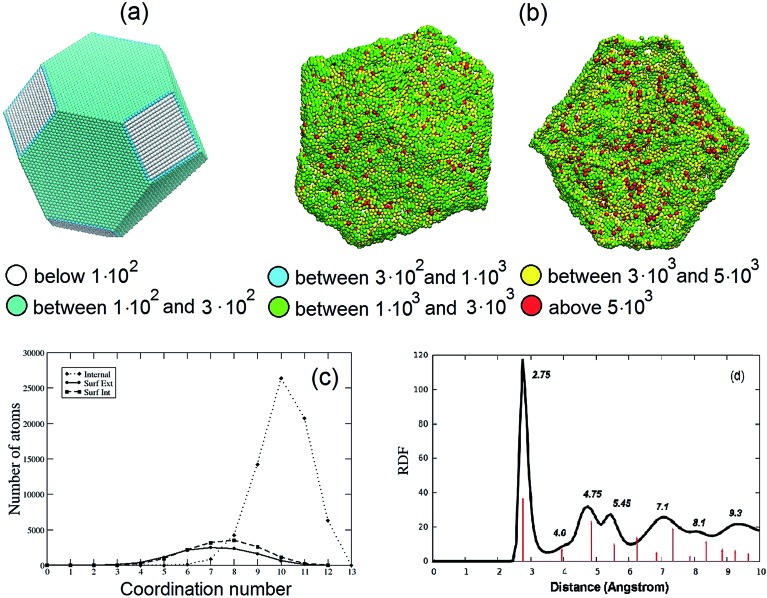
(a and b) Comparison of the atomic stresses in atm nm^3^ at the surface of Pt particles: (a) a truncated-octahedral Pt particle of 98 281 atoms; (b) rear view (left) and cross-section view with only internal and external surface atoms shown (right) of a fully de-alloyed Pt–Ni 30 : 70 10 nm-radius particle (330 000 starting atoms, 97 704 final Pt atoms). Atoms are colored according to the value of the trace of their atomic stress (see legend, note that a positive value indicates tension). (c) Coordination numbers for bulk atoms (dotted line), internal (dashed line) and external (solid line) surface atoms of the same particle shown in (b). (d) Pt–Pt radial distribution function for the same particle shown in (b). The red lines indicate the position of the peaks for the Pt–Pt radial distribution function of the truncated octahedral particle in (a) (reported in full in the ESI†).

The stress analysis of a Pt_3_Ni_7_ particle with an initial 10 nm radius is depicted and contrasted with a regular fcc particle of approximately the same size in Fig. 2(a) and (b). The atomic stress (defined *via* the virial theorem^19^ as the square root of the trace of the tensor of atomic stresses to the second power) is illustrated for a fully de-alloyed Pt–Ni 30 : 70 particle of about 100 000 atoms and compared with that of a truncated-octahedral Pt particle of approximately the same size. From Fig. 2(a) and (b) it is evident that the atomic stresses on the surface atoms of the regular truncated octahedron are at least an order of magnitude smaller than those on the fully de-alloyed Pt_3_Ni_7_ particle, suggesting that complex bonding patterns in the de-alloyed particle lead to strain at the surface (which plays a role in the catalytic behavior, see below).

To provide further structural information, Fig. 2(c) plots the coordination number (*i.e.*, the number of first-neighbors) of bulk, internal and external surface atoms for the same fully de-alloyed Pt_3_Ni_7_ particle. Two points are noteworthy:

(1) all atoms are under-coordinated – around coordination number 6–9 for surface atoms and 10–11 for bulk ones;

(2) no major difference is found between internal and external surface atoms, suggesting homogeneity of the de-alloyed particle surfaces.

Finally, in Fig. 2(d) the Pt–Pt radial distribution function is reported for the same particle. The first-neighbor peak around 2.75 Å is 1% smaller than the 2.775 Å Pt–Pt distance of bulk Pt, which seems reasonable given the reduced coordination. Moreover, peaks corresponding to more distant neighbors can also be inferred, although not as well defined as in the crystal. A comparison of the Pt–Pt radial distribution functions for the truncated octahedral particle of Fig. 2(a) and the nanoporous particle of Fig. 2(d) is also reported in the ESI.† In the experiment, the average nearest-neighbor distance in Pt_3_Ni_7_ prior to de-alloying is 2.62 Å based on the experimental^1^–^3^ lattice parameter of 3.71 Å, which increases to a nearest-neighbor distance of 2.72 Å in the de-alloyed system.

An analysis of structural features as a function of initial Pt–Ni composition is reported in Fig. S1 of the ESI† and in Table 1, which reports the total number of surface atoms (sum of internal and external ones) and the corresponding total surface area per gram of Pt in units of m^2^ g_Pt_^–1^ as a function of the initial Pt–Ni composition for the de-alloyed particles obtained from the 10 nm-radius truncated octahedra. The predicted surface area of *e.g.* 46.5 m^2^ g_Pt_^–1^ for fully dealloyed Pt_3_Ni_7_ is within the range of experimentally measured values (from 38 to 48 m^2^ g_Pt_^–1^).^20^,^21^ Combining this information with the observation noted above that the internal pores of the nanoparticles starting with compositions too rich in Pt are not accessible to reactant molecules because of pore constrictions, we conclude that the catalytically active surface area is roughly doubled at around 30 : 70 Pt–Ni composition with respect to 50 : 50 composition, in fair agreement with experiment.^2^,^3^,^22^ It is also important to observe that for a fully de-alloyed Pt_3_Ni_7_ particle obtained from a truncated octahedron of slightly smaller size (∼8 nm initial radius, shown in the ESI†) the percent of surface atoms is around 22%, which is smaller than that (25.5%) for the larger 10 nm initial radius particle. This counterintuitive result (the percent of surface atoms should correlate inversely with particle size) suggests that there is also an optimal initial nanoparticle size in addition to an optimal initial composition. Indeed this agrees with experimental results on nanoparticle size optimization,^20^ which find a maximum in surface area for nanoporous particles around 8 nm in radius (also depending on the preparation protocol: in ref. 2 the optimal particle size is around 7 nm in radius). This prediction that accessible surface area is reduced if the particle is too small is fully consistent with available experimental information showing that there is a critical size limit below which nanoporosity is reduced or absent *tout court*.^20^,^37^,^40^,^46^

**Table 1:** Geometrical analysis of Pt nanoporous particles obtained by fully de-alloying Pt–Ni truncated octahedra with radius of 10 nm as a function of the initial Ni% content. These results are the average for 5 independent constructions for each composition

Initial % composition in Ni	Number of Pt atoms (radius, nm)	Number of external surface atoms (%)	Total number of surface atoms (%)	Number of rhombi on the surface	Total surface area (m^2^ g^–1^)
80%	64 163 (7.0)	5484 (9%)	22 864 (36%)	16 810	65.0
75%	80 808 (7.2)	7750 (10%)	20 061 (25%)	11 632	45.3
70%	97 704 (7.7)	10 878 (11%)	24 948 (25%)	14 334	46.5
65%	113 727 (7.9)	11 784 (10%)	22 960 (20%)	11 119	42.8
60%	130 111 (8.4)	12 268 (9%)	27 103 (21%)	11 658	38.0

Before leaving the structural analysis, it is important to underline that the nanoporous surfaces produced in our simulations are rather smooth, with a very limited number of steps and kinks, as illustrated in Fig. S3 of the ESI.† Instead we find large surface areas and a huge number of active sites. These active sites have rhombi similar to those on Pt(111) (see the definition of rhombi below) but with noticeably lower coordination. We expect these smooth surface arrangements to be less prone to disaggregation or oxide-formation phenomena, making them robust under the ORR reaction conditions. This is consistent with the massive number of catalytically active sites implied by the experimental rates. The most frequent defects we find are single surface adatoms, which are likely not stable in the harsh conditions of ORR electrochemistry, so that they will be dissolved as the reaction proceeds. This contrasts with a current paradigm in which adatoms, steps and kinks are expected play an important role in ORR.^23^,^24^

### 2.c Surface character and implications for ORR efficiency

**Fig. 3:**
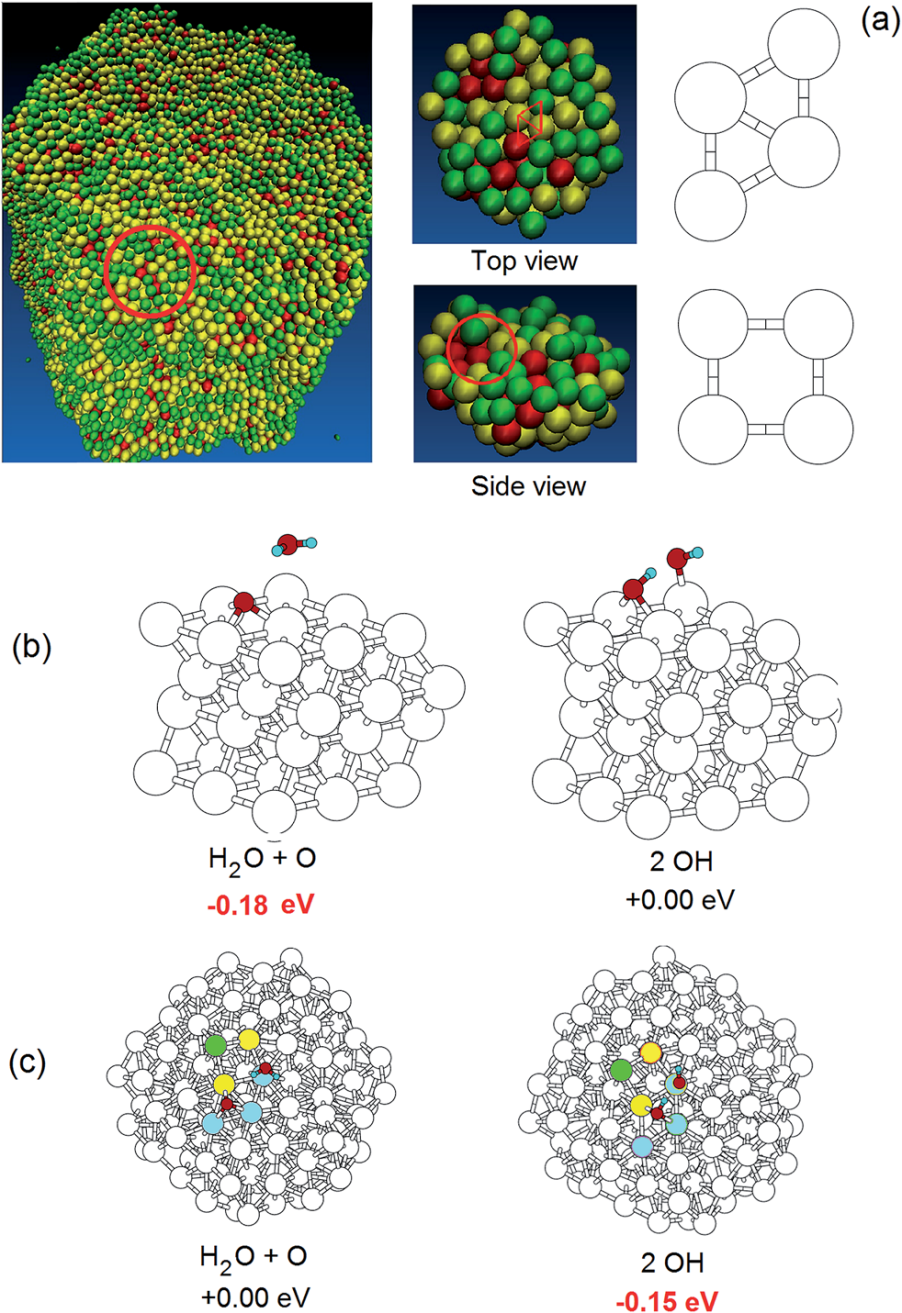
(a) Views of a nanoporous particle obtained by fully de-alloying the 10 nm-radius Pt_3_Ni_7_ nanoalloy (left), top and side views of the finite cluster model extracted from the same nanoparticle and used in the DFT ORR simulation (center) with an example of a rhombus highlighted in red, and definition of a surface rhombus and a surface square (right) – color coding according to atomic stress, as in Fig. 2 – green: low stress, yellow: medium stress, red: high stress. (b) The two competing configurations of the ORR oxygen hydration step on Pt(111) with the relative energetics.^25^ (c) The two competing configurations of the ORR oxygen hydration step with the relative energetics on the finite cluster model shown in (a) (middle).

Table 1 also reports a geometric analysis of Pt–Ni de-alloyed surfaces in terms of rhombi. A rhombus is an ensemble of 4 atoms arranged as two equilateral triangles sharing one edge (depicted in Fig. 5(a), top right). This is distinguished from a square – depicted in Fig. 3(a), bottom right – by having one diagonal larger than the other one, thus resembling the triangular tessellation of the fcc(111) surface. We recall that the Pt(111) surface is more active in ORR than other compact fcc surfaces such as (100),^25^ because radical species such as an oxygen adatom (important intermediates in the ORR) adsorb too strongly on the (100) surface, thus increasing reaction energy barriers of the successive steps. Indeed we calculate an O adsorption energy to be 3.6 eV on Pt(111), and 4.1 eV on Pt(100). Thus a rhomboidal surface arrangement is expected to be advantageous for efficient ORR kinetics. We focus on (111) and (100) patterns because these are commonly encountered on our nanoporous surfaces. Other motifs such as (110), that could be even more advantageous to ORR,^25^ occur very rarely and can be neglected. Table 1 shows that the number of surface rhombi is maximum for 30 : 70 and 20 : 80 initial Pt–Ni ratios, whereas it is appreciably smaller for the other compositions. This suggests that surface geometric features favorable for ORR on nanoporous particles peak around 30 : 70 Pt–Ni initial composition. These studies also suggest that 20 : 80 might also lead to good activity. The problem here may be the stability of the cluster, which we find falls apart for high (≥85%) initial Ni content, and appears not so stable for 80%.

We are now in a position to study relationships between structure and ORR catalytic activity of nanoporous systems. We focus initially on the oxygen hydration (or inverse of a hydroxyl disproportionation) step:

1 O_ads_ + H_2_O_ads_ → OH_ads_ + OH_ads_

where O_ads_, H_2_O_ads_, and OH_ads_ are an oxygen adatom, an adsorbed water molecule, and an adsorbed hydroxyl radical on the Pt surface, respectively, see Fig. 3(b). In previous work we established^25^ that the oxygen hydration (1) is the rate-determining-step (rds) of the ORR in water, which has a significantly lower barrier than O_2_ dissociation – often assumed in previous studies to be the rds for ORR. This is because the large energy gained by hydrating the resulting oxygen adatoms reduces the barrier to break the strong oxygen–oxygen bond. Protonation of the hydroxyl leading to water formation (*i.e.*, the last step of the proposed ORR mechanism) also has a low energy barrier,^25^ so that the rds on the regular fcc(111) Pt surface is the O-hydration step (1) shown in Fig. 3(b), on which we focus for our analysis. The present approach improves upon ORR studies based only on energetics,^50^ due to the subtle dependence of ORR reaction energy barriers and thus kinetics on detailed structural features (*vide infra*), which makes extrapolations based on thermodynamic quantities of uncertain validity.

On Pt(111), O-hydration is endothermic (energetically disfavored) by 0.18 eV, with an energy barrier in the gas phase of ≈0.23 eV. Solvation effects increase the reaction energy difference to ≈0.4 eV, and increase the energy barrier to ≈0.51 eV.^25^,^27^ Of course, protonation of an oxygen adatom to produce an adsorbed hydroxyl could occur in acidic media directly *via* a hydronium ion from the solution phase. We examined this previously,^26^ considering theO_ads_ + H_3_O^+^ + e^–^ → OH_ads_ + H_2_Opathway while explicitly including the presence of an electrode potential and allowing the electron and proton to be decoupled. We found the reaction barrier to be 0.8 eV for this oxygen protonation process, which is much higher than that of the O-hydration path. Superacids from ionomers might also contribute a H^+^ species, but this is unlikely due to their distance from the catalytic surface.

We now want to compare the ORR catalytic activity of fully de-alloyed particles with that on the regular fcc(111) Pt surface *via* accurate density-functional theory (DFT) calculations. To make the problem computationally tractable, we adopt the following Local Embedded Cluster Re-optimization (LECR) procedure:

(1) select a specific rhombus on the surface of a nanoporous particle,

(2) cut a finite cluster around this rhombus by including all atoms closer than a given distance from the center of the rhombus,

(3) freeze the coordinates of all atoms whose bonds have been cut (*i.e.*, those at the boundary with the rest of the original particle),

(4) relax the atomic coordinates of the other atoms using DFT, and

(5) determine the energetics of the O-hydration step on the given rhombus.

We applied this LECR procedure to 4 representative rhombi on each fully de-alloyed particle, thus sampling their catalytic activity with a modest computational effort. Details of the DFT calculations^28^,^29^ are given in the ESI.† Typically the total number of atoms in the finite cluster is ∼150. The results reported below are converged with respect to the distance threshold that defines the finite cluster to make sure that boundary effects due to cutting an embedded region do not affect the results. A pictorial example of the outcome of this procedure is shown in Fig. 3(a) and (c), and the results of these calculations are reported in Table S1 of the ESI† for external surface rhombi of three nanoporous particles generated from Pt–Ni nanoalloys with initial Pt–Ni compositions: 30 : 70, 25 : 75, 20 : 80 (for each rhombus we report two numbers as ORR can occur on its two sides).

The key conclusion from these calculations is that the O-hydration reaction, which is the rds for ORR, becomes strongly more favorable (by 0.2 to 0.6 eV) on the de-alloyed Pt–Ni clusters. The more favorable energetics of O-hydration lead to smaller energy barriers and thus faster kinetics. Indeed, we evaluated several O-hydration reaction pathways using the Nudged Elastic Band (NEB)^30^ method at the DFT level employing 7 images between the initial and final configurations, and found that the calculated energy barrier decreases from 0.23 eV (the value on the Pt(111) surface), to between 0.05 and 0.16 eV while the reaction energy becomes negative by 0.1 to 0.4 eV in contrast to the 0.18 eV positive change for Pt(111).

This unexpected improvement in ORR kinetics is not associated with the presence of steps or other asperities,^22^–^24^ because the surfaces of our nanoporous particles are very smooth. Nor does it arise from electronic effects due to the residual presence of some electropositive element,^20^,^23^,^31^ because in our systems all Ni has been removed. Our more detailed analysis indicates that there exists a correlation between O-hydration energetics and two key structural indicators for the atoms in the rhombi:

(i) average stress (see the ESI† for more details), and

(ii) crystalline character of the bonding environment, as derived from a Common Neighbor Analysis.^32^

**Fig. 4:**
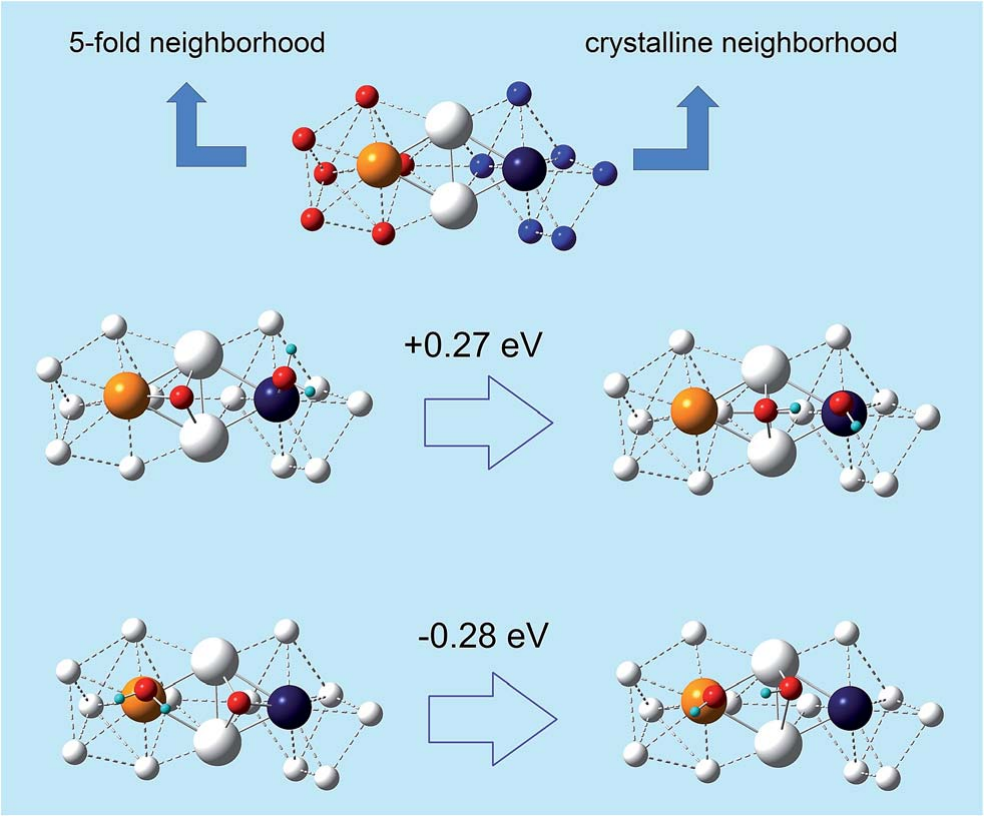
(Top) A view of a surface rhombus of 4 Pt atoms (larger spheres) with their first-neighbors (smaller white spheres). The tip atoms of the rhombus are5-fold-like on the left and fcc-like on the right. This structure is extracted from the surface of a fully de-alloyed Pt_3_Ni_7_ nanoporous particle in which it is embedded (the other atoms of the particles are not shown for clarity). (Middle and Bottom) Energetics of the O-hydration step of the ORR on the left-hand-side (Middle) or right-hand-side (Bottom) of given rhombus.

In other words, the O-hydration step becomes increasingly favorable as the atoms in the rhombi are more stressed but with *less icosahedral-like* character in their bonding environment (or *more crystalline-like*). Since point (ii) is novel and unexpected, we illustrate it in a paradigmatic example in Fig. 4, where a rhombus extracted from a de-alloyed surface derived from a fully de-alloyed Pt_3_Ni_7_ nanoporous particle is shown. Here we see that the two tip atoms exhibit a completely different coordination environment: 5-fold on the left-hand side and fcc-like on the right-hand side. Despite the similarity between the atomic stress values of the tip atoms: 2.8 atm nm^3^ for the left atom and 2.4 atm nm^3^ for the right atom, the sign of the O-hydration energetics on the two triangle of the rhombus is *opposite* by approximately the same amount, thus showing that the 5-fold-like (ORR-unfavorable) or fcc-like (ORR-favorable) is associated with the coordination environment.

To complete our analysis, we take the fcc-coordination-like site on the right-hand side of the rhombus shown in Fig. 4, and consider all the needed steps of the ORR, including the reaction energy barriers evaluated *via* NEB calculations. The Sabatier principle warns in fact that, although we have demonstrated that O-hydration is favored on stressed, under-coordinated and crystalline-like sites of the nanoporous surfaces, the other steps of the ORR – namely, O_2_ dissociation and water formation:

2 O_2ads_ → O_ads_ + O_ads_

**Fig. 5:**
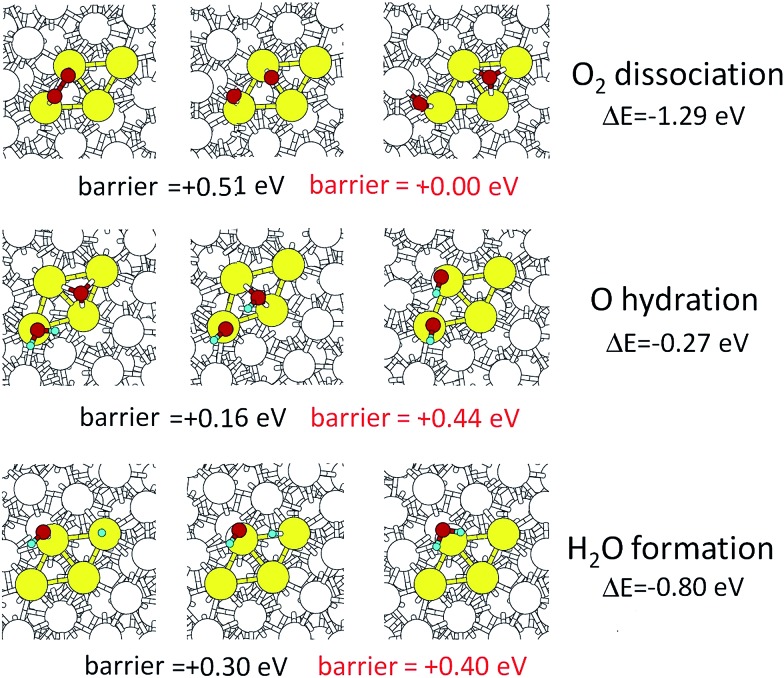
Energetics and pictorial illustration of the three ORR steps on a selected site of Pt_3_Ni_7_ nanoporous particle surface (see text for more details). The initial configurations are on the left-hand side, while the transition states and the products are reported in the middle and right-hand sides, respectively. The thermodynamic reaction energies (Δ*E*) are reported in the right-hand-most column. Below the pictures, the energy barriers in the gas phase are reported in black color and the estimated energy barriers in water are reported in red color.

3 OH_ads_ + H_ads_ → H_2_O_ads_

might increase their reaction energy barriers to become rate-determining. Fig. 5 reports the energetics and a pictorial view of all the three essential ORR steps on the chosen surface site. The barriers for the three steps in the gas phase are in black color in Fig. 5, while we report in red color the final values of the barriers after adding the contribution due to solvation estimated from the corresponding steps on the Pt(111) surface.^25^,^27^ We have not yet been able to carry out accurate calculations of solvation effects in such huge particles but we expect that the solvation contributions to the energy barriers are similar since the mechanisms and reaction paths are similar.

The gas phase O_2_ dissociation step on our rhombus site has a barrier of 0.51 eV which is lower than the 0.56 eV barrier on Pt(111). Since highly polarizable solvents such as water^25^,^27^ reduce this barrier sufficiently for Pt(111) that it is not rate determining we expect the same for our de-alloyed Pt.

For the de-alloyed Pt we find an O-hydration barrier of 0.16 eV in the gas phase, which is 0.07 eV lower than on gas phase Pt(111). We assume that this will increase to 0.44 eV in water, which is smaller by 0.07 eV than the 0.51 eV for this step on Pt(111). We emphasize that in Fig. 5 we chose the site in which O is most strongly adsorbed so that the hydroxyl has to move from a bridge site to an on-top site during ORR. Of the sites that we investigated this site presents the highest O-hydration barrier among the ones exhibiting a favorable thermodynamic reaction energy. Thus we consider the 0.44 eV barrier to be an upper bound.

Finally, we predict that the water formation step has a barrier of 0.30 eV in the gas phase, which is larger than the 0.14 eV barrier it has on Pt(111). However, after adding a 0.10 eV contribution due to solvation,^25^,^27^ to obtain a barrier of 0.40, it does not become rate determining. We conclude that for the fully de-alloyed Ni–Pt particles the barrier for the overall ORR decreases from ∼0.51 eV to 0.44 eV, which at 70 °C operating temperature would lead to a rate increase by a factor of ∼10.

## 3. Discussion

In order to put the present results in the light of previous knowledge, it is useful to briefly recall existing literature. It should be stressed that a variety of different Pt–M systems with different morphological and structural features and different electropositive M element have been investigated. These can be distinguished into three main groups: (i) regular extended surfaces of Pt–M alloys; (ii) nanoporous particles made of alloyed Pt; (iii) finite particles or films made by alloyed Pt and subjected to leaching in order to promote formation of voids or pores. We examine in more detail these three categories:

(i) A series of Pt_3_M (M = Ni, Co, Fe, Ti, V) regular crystalline surfaces have been investigated as ORR catalysts^7^ and interpreted according in terms of a volcano curve using the average d-band position as the descriptor.^33^ The Pt_3_Ni(111) surface, in particular, is found to be 10-fold more active for the ORR than the corresponding Pt(111) surface.^22^ In this alloy, the near-surface layer exhibits a highly structured compositional oscillation in the outermost and third layers, which are Pt-rich, and in the second atomic layer, which is Ni-rich. It should be taken into account, however, that under realistic ORR conditions these extended surfaces transform into nanoporous films,

(ii) Moving from extended surfaces to finite, yet crystalline systems, Pt_3_M (where M = Fe, Ni, or Co) nanoparticles were synthesized;^23^,^34^ they also exhibited high catalytic activities for ORR, with improvement factors of up to 10 *versus* conventional Pt/carbon catalyst. Another possibility is the deposition of a single Pt monolayer on particles made by pure or alloyed transition metals; Adzic and co-workers have investigated this possibility and found that this strategy can enhance catalytic activity towards ORR as interpreted in terms of inducing lattice strain in the outermost layer^35^ or shifting d-band centre.^36^

(iii) In the last decade, much attention has been devoted to systems obtained starting from Pt_1–*x*_M_*x*_ particles or films (with *x* > 0.5 and M = Mn, Fe, Co, Ni, Cu) and subjecting them to an electrochemical treatment leading to dealloying and leaching of the electropositive M component.^1^–^3^,^8^,^20^,^35^,^37^–^40^,^49^ These particles, often referred as spongy or porous, achieve at the same time a reduction of used Pt and an improvement of catalytic activity by more than 1 order of magnitude *versus* conventional pure Pt catalysts. These systems are very complex, as the nature of the alloy, the initial composition and the experimental procedure used in the dealloying procedure play a crucial role in determining the final morphology and the metal coordination environment of the final particles.^39^ Due to this complexity, general rules that correlate catalytic activity to microscopic structure are still missing, as well as predictive physical descriptors to design optimal ORR catalysts. Two main points can nevertheless be highlighted: (i) *the nature of the metal can correspond to a different onset on the size of pore-formation*; Oezaslan *et al.*^37^ have shown that, when using M = Co or Cu and *x* = 0.75, voids are exhibited for particles with a diameter larger than 30 nm, whereas Snyder *et al.*^20^ have shown that, when using M = Ni at the same composition of *x* = 0.75, the on-set in the formation of nanoporous particles takes place at a diameter of about 15 nm with a maximum catalytic activity for a diameter between 15 and 20 nm (as mentioned above there is a critical size limit below which nanoporosity is reduced or absent *tout court*); (ii) *different leaching procedures on the same alloy and on particles of similar size can affect both the morphological shape and the atomistic coordination environment* of the final particles and hence their catalytic activity: Gan *et al.*,^40^ who investigated Ni–Pt alloyed particles in a size range between 7 and 26 nm in diameter, found that leaching in oxidizing conditions led to the formation of nanoporous particles with a reduced activity (ascribed by the authors to reduction of surface strain) with respect to non-porous particles leached according to a non-oxidizing protocol. A non-oxidizing protocol was used by Snyder *et al.* on the same Ni–Pt alloy^20^ to get nanoporous particles of 15–20 nm in diameter with an enhanced catalytic activity with respect to pure Pt systems; a measurement of the electrochemically active surface area (ECSA) of the obtained particles was performed and the authored ascribed the improved activity to their high surface/volume ratio and to a core–shell, Pt-exoskeleton structure with residual Ni in the centre of the particles. Alternative, although related, morphologies have also been obtained in the form of “concave octahedra” or “nanoframes”. Starting from Ni rich octahedral particles with an initial diameter of about 13 nm in electrochemical environments, Cui *et al.*^41^ have shown that Ni preferentially segregates at (111) facets and it is leached from there forming ‘concave octahedra’ with the final stabilization of particles in form Pt-rich skeleton frameworks. Chen *et al.*^44^ have shown that crystalline PtNi_3_ polyhedra of about 20 nm in diameter transform in solution by interior erosion (*i.e.*, a very slow non-oxidizing protocol) into nanoframes with Pt_3_Ni composition and with both interior and exterior surfaces exhibiting the usual nanosegregated Pt-skin structure and exhibiting a spectacular ORR activity.

With respect to this previous literature, our first-principles-based reaction simulations lead us to conclude that fully de-alloyed (nanoporous) Pt_3_Ni_7_ particles are particularly active for ORR catalysts both because they possess significantly enhanced accessible surface areas and because they present smooth surfaces with a triangular tessellation of under-coordinated (thus stressed) but still crystalline-like bonding environments. This latter feature (that has never been discussed in previous literature) favors hydroxyl formation from hydrolysis of surface oxygen, thus decreasing the energy barrier of the rate determining step for ORR on Pt(111) surface. In our analysis we do not require the presence of subsurface species such as more electropositive elements (Ni),^20^,^23^,^31^ nor do we require the low-coordinated Pt atoms at steps or kinks,^23^,^24^ both which features may be expected to be labile under the harsh ORR conditions. Instead we find that the 30 : 70 composition for the as prepared catalyst is optimum because

· particles with higher initial Pt content have a smaller accessible active surface area,

· particles with lower initial Pt content tend to be mechanically unstable, favoring fragmentation into smaller clusters that are less effective ORR catalysts due to the increased number of 5-fold-coordinated rhombi^42^,^43^ which we predict as less favorable for ORR catalysis.

In excellent tune with existing knowledge,^20^,^40^ these simulations suggest that kinetic effects in the preparation protocol are crucial in maximizing nanoporous catalytic activity:^44^ 5-fold motifs are favored by fast atomic diffusion and rearrangement in the under-coordinated régime, whereas under-coordinated and strained yet fcc-like arrangements must be achieved in a slow atomic diffusion régime in order to promote ORR. It may be that the reason why Co or Mn alloying elements are less effective than Ni (see *e.g.* Fig. 6 in ref. 2) is that their bond strengths with Pt lead to different de-alloying kinetics.^45^

Our simulations also rationalize the experimental observation that there is an optimal particle size (around 8 nm in radius^20^), since we find that catalytic activity is low for small clusters because of increased 5-fold-motifs and decreased total surface area in addition to reduced or *tout court* absent porosity, whereas particles that are too large suffer from a low surface/volume ratio.

Our predictions from RMD simulations are thus basically consistent with existing knowledge. Of course, the experimental situation is considerably more complicated than assumed in our simulations. For example, we did not consider the fact that in real fuel cells electrocatalyst surfaces are in proximity with super-acid ionomers from the proton exchange membrane which can affect the polarization characteristics and composition of the solvent medium (favoring increased concentrations of ionic species or – less likely – providing a source of hydrogen ions). Moreover, as discussed above, we assumed complete removal of Ni, whereas some amount of electropositive elements remains as minority component in the catalyst nanoparticles (note however that experimental determination of residual Ni may also be biased by the presence of deposited Ni^2+^ ions from the solution). Also, our protocol cannot describe the fine details of the experimental corrosion process. Nevertheless, we find good semi-quantitative match between predictions from the simulations and experimental information available on these systems. Our fully de-alloyed Pt_3_Ni_7_ particles exhibit a catalytically active surface area which is ∼ twice that for the 50 : 50 composition (for which internal surfaces are inaccessible to reactants due to nanopores not reaching up to the external surface). This doubling of surface area is in fair agreement with experiment,^2^,^3^,^22^ in which an increase of 50% in electrocatalytically active surface area is often observed, see Fig. 3 and 4 of ref. 2, reaching even higher values in some cases.^8^,^46^ Moreover, for the fully de-alloyed Pt_3_Ni_7_ particles we predict that the gas-phase energy barrier for the ORR rds decreases to ≈0.07–0.16 eV compared with 0.23 eV on Pt(111) (the component due to solvation is expected to be roughly constant).^25^,^27^ This decrease in the energy barrier could account for an average increase in ORR rate by a factor of 10–20 at 70 °C with respect to Pt(111). However we must normalize this increase by the number of rhombi with the proper (fcc-like) coordination and we must consider that the experimental pure-Pt catalysts are more active than the Pt(111) surface. This accounts for an increase in ORR due to geometric effects by a factor of 2, and an overall enhancement of the ORR catalytic activity by probably a factor of ≈4, which is consistent with experiment.^1^–^3^

## 4. Conclusions

We use first-principles-based multiscale simulation tools to explore our hypothesis that the spectacular performance of Pt_3_Ni_7_ particles as ORR catalysts arises from de-alloying. Indeed these simulations provide a theoretical explanation for the puzzling observation of a pronounced maximum in the ORR catalytic activity of Pt–Ni catalysts around 70% Ni initial content, despite the observation that Ni is mostly leached out from the surface layers of the catalysts after ORR cycling.^9^

To test these ideas we first devised a protocol to generate Pt–Ni de-alloyed structures using the ReaxFF reactive force field, then we conducted a detailed analysis of their geometric features in terms of coordination number, atomic stress, crystalline *vs.* 5-fold character of bonding environment, and surface tessellation in terms of ‘rhombi’. Finally we combined this information with DFT calculations of ORR energetics and activation barriers.

A crucial result from the present study is that the fully de-alloyed Pt_3_Ni_7_ particle surfaces exhibit smooth triangulated surface arrangements reminiscent of a regular fcc Pt(111) surface but under-coordinated and stressed in such a way that the barrier of the ORR rate-determining step is reduced significantly. We predict a maximum in catalytically active surface area as a function of size at ∼8 nm radius with a maximum in ORR activity as a function of initial Pt–Ni composition at around 30 : 70. Thus the simulations explain the Pt_3_Ni_7_ paradox.

This atomistic picture of the structures and properties of the fully de-alloyed catalyst particles provides the basis for a deeper understanding of the experimental findings. Moreover it may be useful in suggesting experiments in synthesis aimed at optimization to achieve improved materials with enhanced catalytic activity and selectivity. This suggests future developments in which an explicit simulation of de-alloying kinetics^45^ is explored for ternary alloys^47^ that might leave some alloying atoms in the surface active regions. We could also examine more exotic morphologies,^44^ and coupling with optimization by tuning solvation effects.^27^,^48^

## Supplementary Material

Supplementary information

Supplementary information

Supplementary information

Supplementary information
