# Supplementary material for: The atomistic origin of the extraordinary oxygen reduction activity of Pt3Ni7 fuel cell catalysts
Source: Chem Sci. 2015 Apr 29;6(7):3915–25. doi: 10.1039/c5sc00840a (PMC5707479; doi:10.1039/c5sc00840a)
Supplement: Supplementary file 1 [file SC-006-C5SC00840A-s001.pdf]

Electronic Supplementary Material (ESI) for Chemical Science.
This journal is © The Royal Society of Chemistry 2015

Supporting Information for:
"The atomistic origin of the extraordinary Oxygen Reduction activity of Pt_3Ni_7 fuel cell catalysts"

Alessandro Fortunelli[1,2,*], William A. Goddard III[2,*], Luca Sementa[1], Giovanni Barcaro[1], Fabio R. Negreiros[1], Andrés Jaramillo-Botero[2]

[1]CNR-ICCOM and IPCF, Consiglio Nazionale delle Ricerche, via Giuseppe Moruzzi 1, 56124, Pisa, Italy

[2]Materials and Process Simulation Center, California Institute of Technology, MC 139-74, 1200 E California Blvd, Pasadena, California 91125, United States

[*]Corresponding authors' e-mail:
AF – alessandro.fortunelli@cnr.it, afloer@caltech.edu; WAG – wag@wag.caltech.edu

Here we provide further computational details and additional structural and ORR catalytic information on the Pt-Ni dealloyed particles.

As described in detail in Ref. [7], the ReaxFF parameters used to describe the interactions in Pt clusters was fitted to reproduce a large set of DFT-derived quantities: the equation-of-state for various bulk Pt phases (fcc, bcc, sc, and A-15), the stability of various surface orientations, finite Pt clusters (up to 35 atoms).

In connection with the nanoparticle structure generation protocol, it is important to note that the initial local minimizations of the nanoparticles after Ni removal are performed to avoid disruption of the original random fcc framework. This is important for a proper description of dealloyed nanoporous systems. Thus, at each conjugate gradient step, we limited the maximum difference in Cartesian coordinates to 0.2 Å, until convergence threshold criteria of 4 10^{-6} eV on the energy and 4 10^{-8} eV/Å on the gradient thresholds were met.

For convenience of the reader and further analysis, the Cartesian coordinates of the three particles shown in Fig. 1 of the main text are provided as separate text files with names: Pt5Ni5.xyz, Pt3Ni7.xyz, Pt1Ni9.xyz. These structures have been selected from among the several runs of our structure generation protocols and are representative of typical situations. Further information is available upon request.

Analysis of structural features as a function of initial Ni:Pt composition is reported in Fig. S1 and S2.

In Fig. S1, a comparison of the Pt-Pt radial distribution functions for the truncated octahedral particle of Fig. 2(a) of the main text and the nanoporous particle of Fig. 2(b) of the main text is reported.

In Fig. S2(a), the relative amount of surface external and surface internal atoms is reported, whence it can be appreciated that the percent of surface external atoms is approximately constant in the investigated Ni:Pt range, whereas that of internal surface atoms increases at 80:20 composition. This corresponds to a general observation: many structural indicators for the nanoporous structures evolve smoothly from 50:50 up to 75:25 initial Ni:Pt content, then at 80:20 significant changes occur, preluding cluster fragmentation at Ni:Pt = 90:10.

In Fig. S2(b) the average atomic stress as a function of the Ni initial composition is reported. The average atomic stress is defined as the average over all atoms of the square root of the trace of the tensor of atomic stresses to the second power. An irregular plateau between 65:35 and 75:25 initial composition can be observed.

Also interesting is information on the coordination environment of surface atoms, which we obtain employing a variant of the Common Neighbor Analysis (CNA),[1] aimed at distinguishing between icosahedral-like and crystalline-like bonding environments. We use the following procedure: for each Pt atom of the particle, we define its first-neighbour coordination shell as the set of Pt atoms lying at a distance smaller than 3 Å. Selecting one atom at a time in the first-neighbour shell, we use its bond distance R from the central atom to create a perfect icosahedron of radius R and we optimally superimpose this icosahedron onto the first-neighbor shell, i.e., we determine the orientation of the icosahedron which minimizes the sum of distances between its atoms and the atoms of the first-neighbour shell, neglecting mismatching atoms if the coordination number is less than 12. We repeat this procedure for each first-neighbour atom and report as 5-fold index in Fig. S2(c) the minimum value of the minimized sum of distances. Clearly, the lower the value of the 5-fold index, the more icosahedral-like the coordination environment of the given atom. From previous work[2] it is known that non-crystalline bonding environments such as icosahedral and poly-icosahedral are common in small Pt clusters, so that amorphous configurations commonly found in these systems can be described as originating from a rosette-like transformations of five-fold icosahedral vertexes.[2] Other analyses propose a transition to fcc-like structures at very small sizes, with a dominating role of (111) facets.[3] Fig. S2(c) plots the average 5-fold index signature for surface external and internal atoms separately. This quantity shows an irregular maximum corresponding to less icosahedral character between 65:35 and 75:25 initial composition for the internal surface sites, whereas for the external surface sites (which are of interest for ORR catalysis) the icosahedral character progressively increases with initial Ni content, but interestingly with a steeper gradient from Ni:Pt = 75:25 onward, suggesting a near-optimality of 70:30 initial composition.

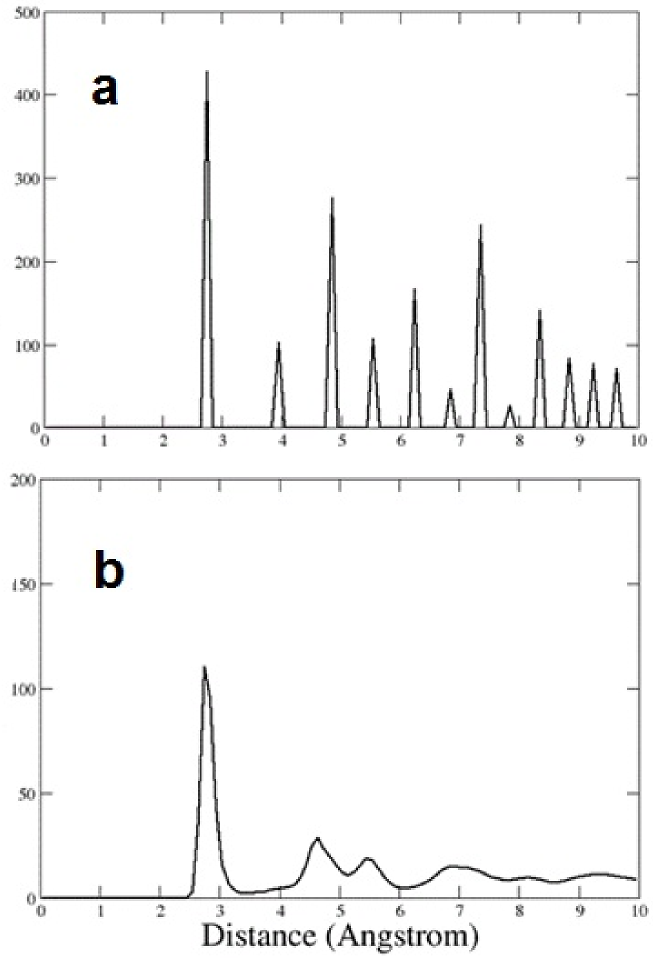

Figure S1: Pt-Pt radial distribution functions for: (a) the truncated octahedral particle of Fig. 2(a) of the main text, and (b) the nanoporous particle of Fig. 2(d) of the main text.

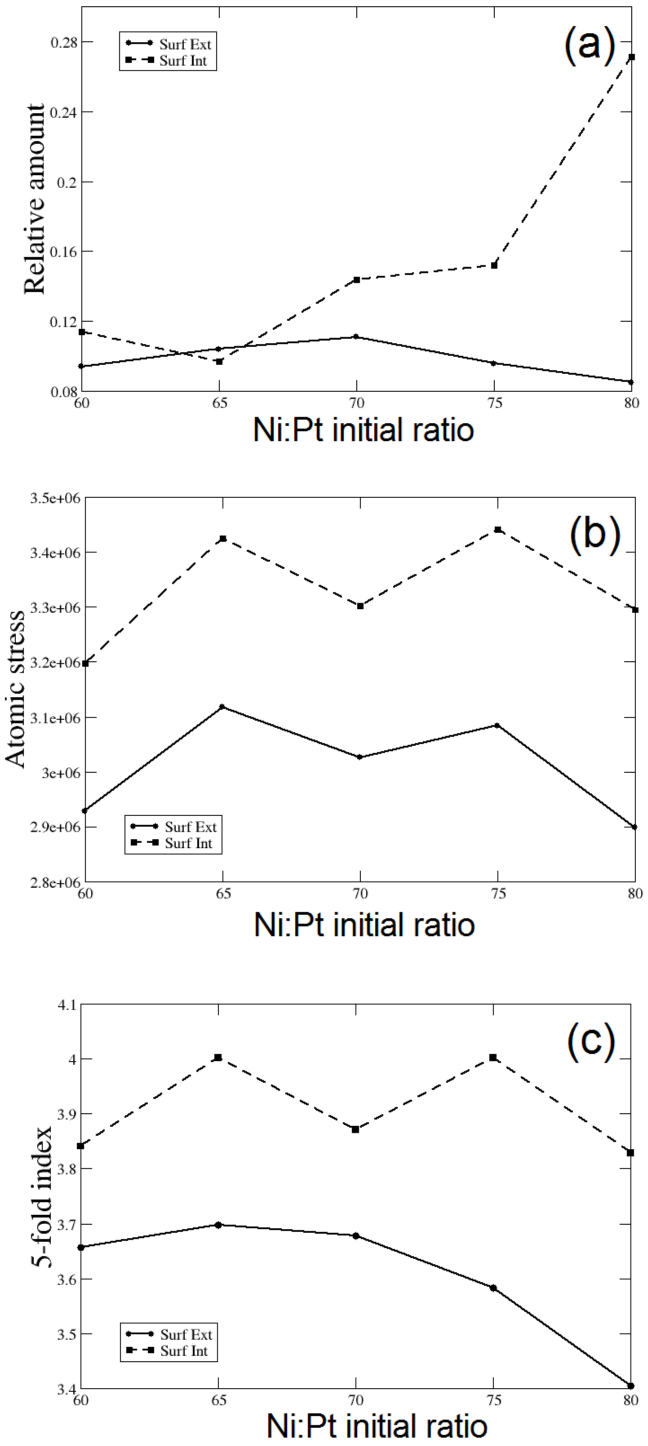

Figure S2: Analysis of nanoporous aggregates generated from Pt-Ni nanoparticles with 10 nm in radius as a function of initial Ni:Pt ratio: (a) relative amount of internal (dashed line) and external (solid line) atoms; (b) evolution of average atomic stress in atm·Å^3 for internal (dashed line) and external (solid line); (c) evolution of average 5-fold index for internal (dashed line) and external (solid line) from a variant of CNA analysis, see text for details.

Finally, we conclude our structural analysis by quantifying the smoothness of the nanoporous surfaces. To this aim, we plot in Fig. S3 the distribution of dihedral angles in the surface rhombi. By defining atoms 1,2 in a rhombus as the farthest ones and atoms 3,4 as those on the diagonal of the rhombus, the dihedral angle is calculated as the angle formed between atoms 1,2 and the middle point between 3 and 4, as illustrated in Fig. S3. This figure clearly shows that in nanoporous particles up to 70:30 Ni:Pt initial composition most dihedral angles lie between 140° and 180°, thus corresponding to smooth particle surfaces and excluding the massive presence of adatoms and surface defects.

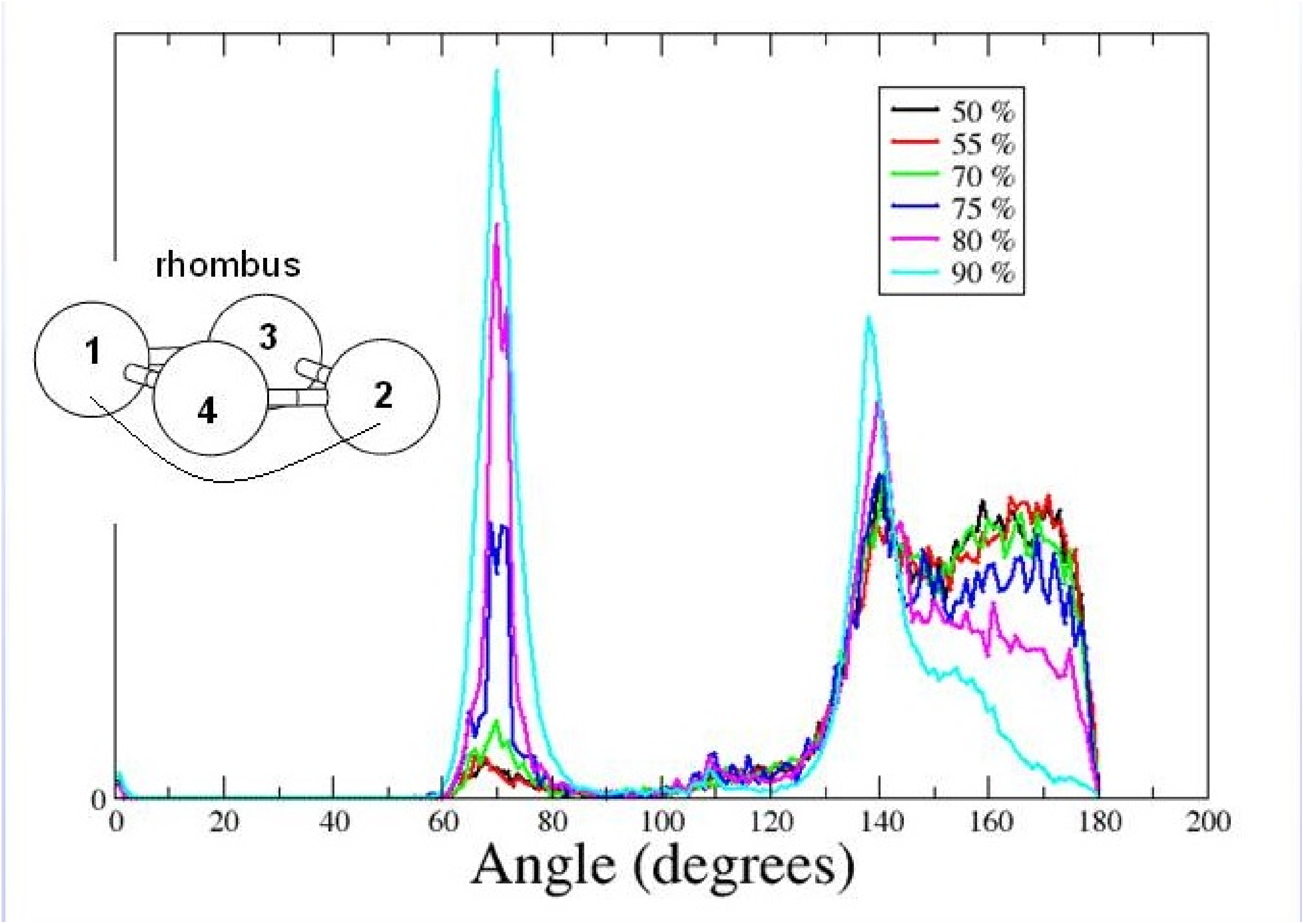

Figure S3. Plots of the distribution of dihedral angles in the surface rhombi, see text for details.

So far we have considered only de-alloyed particles obtained from the initially 10-nm-radius Pt-Ni truncated octahedral. Smaller particles are less interesting, because the nanopores usually do not reach up to the surface, as pictorially illustrated in Fig.S4, where an example of a de-alloyed Pt particles obtained starting from truncated-octahedral Pt-Ni nanoparticle about 8 nm in radius with initial compositions Pt:Ni = 30:70 is shown.

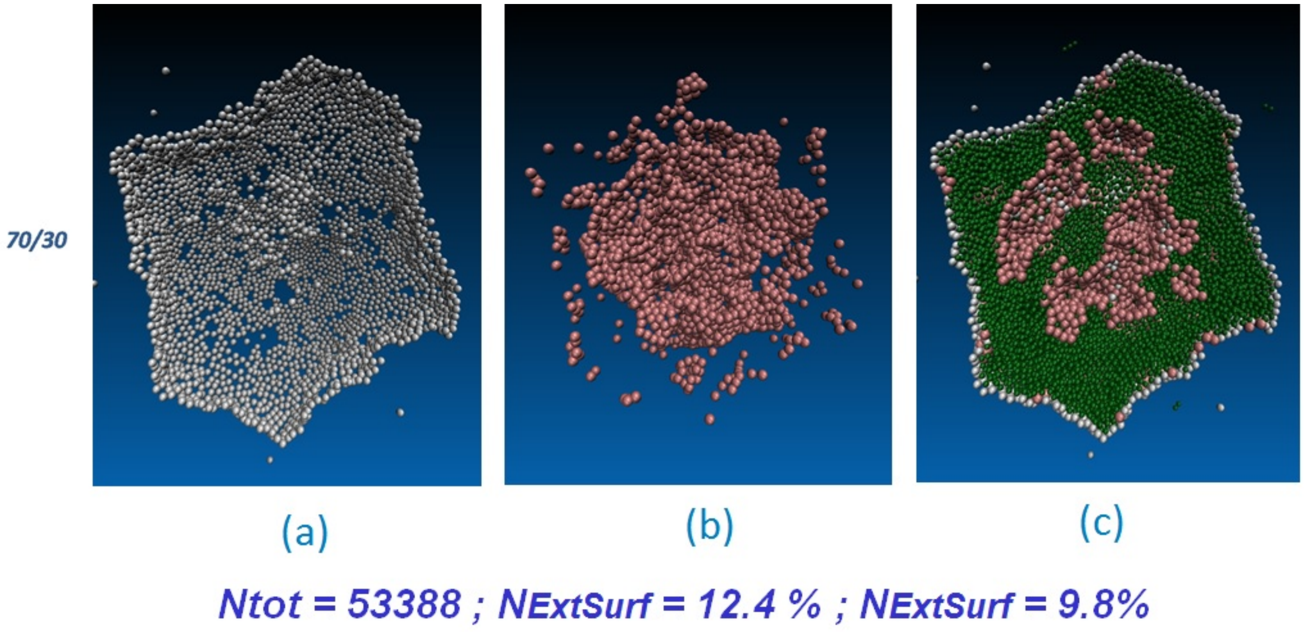

Figure S4. Pictorial views of a de-alloyed Pt particle obtained starting from a truncated-octahedral Pt-Ni nanoparticle (initially about 8 nm in radius and with composition Pt:Ni = 30:70) after complete removal of Ni, followed by minimization and molecular dynamics equilibration at 70 C. For clarity, a cross-section (half) through the Pt particles is shown. (a) atoms on the external surfaces; (b) atoms on the internal surfaces; (c) all atoms. Color and definition of external and internal surfaces atoms as in Fig. 1 of the main text.

Finally we provide further details of the DFT calculations reported in the main text. We used the Quantum-Espresso (QE) package[4] to solve the Kohn-Sham DFT equations, the Perdew-Burke-Ernzerhof (PBE) exchange-correlation functional,[5] a spin-unrestricted framework, ultra-soft pseudopotentials from the QE library, cut-offs of 40 and 400 Ry on the wavefunctions and electronic density, respectively, and a Gaussian smearing of the one electron levels of 0.002 Ry.

We focused on the rate-determining-step (rds) of the ORR in water, as determined in previous work,[6] i.e., the oxygen hydration (or inverse of a hydroxyl disproportionation) step:

$O_{ads} + H_2O_{ads} \rightarrow OH_{ads} + OH_{ads}$ (1)

where O_{ads}, H_2O_{ads}, and OH_{ads} are an oxygen adatom, a water molecule, and a hydroxyl radical all adsorbed onto the Pt surface.

However for one case we also examined the O_2 dissociation and water formation steps, as reported in Figure 5 of the main text.

We single out a few rhombi on the surface of Pt-Ni dealloyed particles as described in the main text, and report in Table S1 selected results of total reaction energy calculations for external surface rhombi of three nanoporous particles generated from Pt-Ni nanoalloys with initial compositions in Pt:Ni: 30:70, 25:75, 20:80. The reported examples are representative of rhombi with a different bonding environment, both in terms of average coordination number and icosahedral character as determined via our variant of the Common Neighbor Analysis.[1] We see that the ORR rds O-hydration reaction, which is energetically unfavorable on Pt(111), can become strongly favored on Ni-Pt dealloyed surfaces.

initial % composition in Ni	site number	Delta E (eV)	initial % composition in Ni	site number	Delta E (eV)
**80 %**	1	**+0.06**	**75 %**	5	**+0.14**
**80 %**	2	-0.35	**75 %**	6	-0.51
**80 %**	3	-0.08	**75 %**	7	-0.90
**80 %**	4	**+0.29**	**75 %**	8	-0.07
**80 %**	5	-0.52	**70 %**	1	-0.16
**80 %**	6	-0.62	**70 %**	2	**+0.01**
**80 %**	7	**+0.27**	**70 %**	3	-0.33
**80 %**	8	-0.28	**70 %**	4	-0.39
**75 %**	1	-0.23	**70 %**	5	**+0.06**
**75 %**	2	-0.42	**70 %**	6	**+0.09**
**75 %**	3	-0.20	**70 %**	7	-0.29
**75 %**	4	-0.36	**70 %**	8	-0.37

Table S1: Energetics of the ORR on selected sites on the external surfaces of nanoparticles obtained by dealloying truncated octahedra with initial radius of 10 nm and different initial Ni content as predicted by DFT calculations. See text for more details.

References

[1] Faken, D.; Jonsson, H. Systematic analysis of local atomic structure combined with 3d computer graphics. *Comput. Mater. Sci.* **1994**, *2*, 279 – 286.

[2] Aprà, E.; Baletto, F.; Ferrando, R.; Fortunelli, A. Amorphization mechanism of icosahedral metal nanoclusters. *Phys. Rev. Lett.* **2004**, *93*, 065502.

[3] Kumar, V.; Kawazoe, Y. Evolution of atomic and electronic structure of Pt clusters: Planar, layered, pyramidal, cage, cubic, and octahedral growth *Phys. Rev. B* **2008**, *77*, 205418.

[4] Giannozzi, P.; Baroni, S; Bonini, N.; Calandra, M.; Car, R.; Cavazzoni, C.; Ceresoli, D.; Chiarotti, G.; Cococcioni, M; Dabo, I.; et al. QUANTUM ESPRESSO: a modular and open-source software project for quantum simulations of materials. *J. Phys.: Condens. Matter*. **2009**, *21*, 395502: 1− 19.

[5] Perdew, J.; Burke, K.; Ernzerhof, M. Generalized Gradient Approximation Made Simple. Phys. *Rev. Lett.* **1996**, *77*, 3865 – 3868.

[6] Sha, Y.; Yu, T.H.; Merinov, B.V.; Shirvanian, P.; Goddard, W.A. Oxygen Hydration Mechanism for the Oxygen Reduction Reaction at Pt and Pd Fuel Cell Catalysts *J. Phys. Chem. Lett.* **2011**, *2*, 572 –576.

[7] Sanz-Navarro, C.F.; Astrand, P.O.; Chen, D.; Ronning, M.; van Duin, A.C.T.; Jacob, T.; Goddard, W.A. Molecular Dynamics Simulations of the Interactions between Platinum Clusters and Carbon Platelets. *J. Phys. Chem. A* **2008**, *112*, 1392 – 1402.
